# Specific genetic aberrations of parathyroid in Chinese patients with tertiary hyperparathyroidism using whole-exome sequencing

**DOI:** 10.3389/fendo.2023.1221060

**Published:** 2023-10-03

**Authors:** Lei Li, Qixuan Sheng, Huajin Zeng, Wei Li, Qiang Wang, Guanjun Ma, Xinyun Xu, Ming Qiu, Wei Zhang, Chengxiang Shan

**Affiliations:** ^1^ Department of Thyroid, Breast and Hernia Surgery of Changzheng Hospital Affiliated with Naval Military Medical University, Shanghai, China; ^2^ Department of General Surgery, Shanghai General Hospital, Shanghai Jiao Tong University School of Medicine, Shanghai, China

**Keywords:** tertiary hyperparathyroidism, whole-exome sequencing, somatic mutation, driver mutation, copy number variants

## Abstract

**Background:**

Tertiary hyperparathyroidism (THPT) is a peculiar subtype of hyperparathyroidism that usually develops from chronic kidney disease (CKD) and persists even after kidney transplantation. Unlike its precursor, secondary hyperparathyroidism (SHPT), THPT is characterized by uncontrolled high levels of calcium in the blood, which suggests the monoclonal or oligoclonal proliferation of parathyroid cells. However, the molecular abnormalities leading to THPT have not yet been fully understood.

**Methods:**

In this study, we analyzed DNA samples from hyperplastic parathyroid and corresponding blood cells of 11 patients with THPT using whole-exome sequencing (WES). We identified somatic single nucleotide variants (SNV) and insertions or deletions variants (INDEL) and performed driver mutation analysis, KEGG pathway, and GO functional enrichment analysis. To confirm the impact of selected driver mutated genes, we also tested their expression level in these samples using qRT-PCR.

**Results:**

Following quality control and mutation filtering, we identified 17,401 mutations, comprising 6690 missense variants, 3078 frameshift variants, 2005 stop-gained variants, and 1630 synonymous variants. Copy number variants (CNV) analysis showed that chromosome 22 copy number deletion was frequently observed in 6 samples. Driver mutation analysis identified 179 statistically significant mutated genes, including recurrent missense mutations on TBX20, ATAD5, ZNF669, and NOX3 genes in 3 different patients. KEGG pathway analysis revealed two enriched pathways: non-homologous end-joining and cell cycle, with a sole gene, PRKDC, involved. GO analysis demonstrated significant enrichment of various cellular components and cytobiological processes associated with four genes, including GO items of positive regulation of developmental growth, protein ubiquitination, and positive regulation of the apoptotic process. Compared to blood samples, THPT samples exhibited lower expression levels of PRKDC, TBX20, ATAD5, and NOX3 genes. THPT samples with exon mutations had relatively lower expression levels of PRKDC, TBX20, and NOX3 genes compared to those without mutations, although the difference was not statistically significant.

**Conclusion:**

This study provides a comprehensive landscape of the genetic characteristics of hyperplastic parathyroids in THPT, highlighting the involvement of multiple genes and pathways in the development and progression of this disease. The dominant mutations identified in our study depicted new insights into the pathogenesis and molecular characteristics of THPT.

## Introduction

Hyperparathyroidism (HPT) is a common endocrine disorder, characterized by aberrant parathyroid gland enlargement, excessive circulating parathyroid hormone (PTH), and disturbed bone and mineral metabolism. Tertiary hyperparathyroidism (THPT), a complex and unique subtype of HPT (1, 2), arises predominantly from secondary hyperparathyroidism (SHPT) during the prolonged course of chronic kidney diseases (CKD) and continues even after kidney transplantation, while showing minimal response to therapeutic agents (3).

In contrast to SHPT, which is its precursor, THPT is characterized by uncontrolled hypercalcemia that resembles the autonomous form of HPT. This suggests monoclonal or oligoclonal proliferation of parathyroid cells in THPT, rather than a widespread hyperplastic expansion of all parathyroid cells in response to external growth stimuli. Although X-inactivation assays have already shown that monoclonal outgrowths can occur against a backdrop of supposed generalized non-clonal parathyroid hyperplasia in the context of CKD (4), the pathogenesis of THPT remains incompletely defined.

To fully understand the development of parathyroid tumors caused by clonal growth, it is important to identify the potential acquired and selected driver mutations involved in the process. In cases of nonhereditary or sporadic parathyroid adenomas (PAs), specific mutations in certain genes had been shown to be responsible for their development. Two genes, MEN1 and CCND1, an archetype tumor suppressor and a prototypical proto-oncogene, respectively, had been unequivocally established as paramount tumorigenic drivers in PAs (5, 6). MEN1 was frequently associated with somatic mutations, occurring in 12% to 35% of sporadic PAs, while rearrangements of the CCND1 locus appeared to occur in up to 8% of PAs (7). Additionally, novel ZFX mutations at residues p.R786 and p.R787 were discovered in 4.6% of a parathyroid adenoma cohort (8, 9). Moreover, confirmed somatic mutations of CDNK1B were observed in less than 1% of sporadic PAs (10, 11). Still, other genes, some involved in pathways leading to MEN1 inactivation (12), CCND1 activation (13), WNT activation (14), or the PTH dysregulation (15), had been and continued to be explored as candidate parathyroid tumor driving genes. All of these findings could provide alternative potential targets for the treatment of PAs.

Despite the vast knowledge on genetic alterations in sporadic primary hyperparathyroidism (PHPT), the underlying molecular mechanisms of THPT remained largely elusive, particularly in the Chinese population. To bridge this gap, our study employed a comprehensive approach utilizing whole-exome sequencing (WES) analysis of matched tumor and blood cell DNA samples from Chinese THPT patients. We aimed to identify potential genetic aberrations implicated in the pathogenesis of THPT and to assess the mutation frequency of established genes in the Chinese cohort.

## Materials and methods

### THPT diagnostic criteria

To fulfill the diagnosis of THPT, a triad of conditions must be met according to the following established criteria (16): (i) A documented history of renal insufficiency concomitant with SHPT, either prior to or after renal transplantation; (ii) Elevated circulating levels of PTH that are biochemically abnormal, concomitant with uncontrolled hypercalcemia; (iii) Appearance of abnormal enlargement of parathyroid glands, in conjunction with pathologically confirmed nodular or adenomatous hyperplasia.

### Clinical samples

The study set consisted of DNA samples isolated from pathological parathyroid hyperplasia tissue, as well as matched blood samples from each patient, following standard procedures. Agarose gel electrophoresis was employed to analyze the extent of degradation and the presence of RNA contamination. The purity of the extracted DNA was evaluated using Nanodrop, and the DNA concentration was precisely measured using Qubit. Samples over 1.5ug in contents as well as with an optical density (OD) value ranging from 1.8 to 2.0 were selected for further analysis.

The study protocol was approved by the Committee of Ethics Review Board of Changzheng Hospital affiliated to Naval Military Medical University. All patients provided informed consent for inclusion in the study according to the guidelines at our institution.

### Whole-exome library construction and high-throughput sequencing

Whole-exome of the DNA samples was captured and enriched strictly deferring to the protocol of commercially available Agilent SureSelect v6 Kit on Agilent Liquid Chip Capture System. Specifically, prepped DNA fragments were hybridized with biotinylated RNA library “baits”, and then mixed with streptavidin coated magnetic beads. Beads-conjunct fractions were selectively captured by magnet and put to washing and digestion of RNA “baits” subsequently. The fractions obtained were then amplified by PCR for the following sequencing. Captured libraries were firstly testified for its quality including insert size and effective concentration by Agilent 2100 and Q-PCR respectively. Qualified libraries were sequenced on illumina Novaseq 6000 sequencing platform with reads length of 150bp. Raw image data were converted into raw sequence reads through Base Calling analysis.

### Bioinformatics analysis

Sequencing data were processed for base quality distribution and GC content distribution by statistical methods. Raw reads were filtered out of reads with adapters or with low quality to get clean reads, which were mapped to the reference genome Hg 19 using BWA (http://bio-bwa.sourceforge.net/) software. Reads generated by PCR-duplication were discarded using Picard software. Analysis of the raw data from WES was performed according to GATK Best Practices recommendations (www.broadinstitute.org/gatk/). Single nucleotide variants (SNV) and insertions or deletions (INDEL) variants detection and filtration were performed using the genome analyzer Mutect2 software (17). Copy number variants (CNV) were analyzed by CNVkit (http://github.com/etal/cnvkit), and structural variants (SV) analyzed by Manta (https://github.com/Illumina/manta). The identified somatic variants were further annotated using VEP software (18). Functional impact predictions of each verified somatic variant were performed using both Polyphen2 (http://genetics.bwh.harvard.edu/pph2/) and SIFT (http://sift.jcvi.org). Driver mutations were analyzed by OncodriveCLUSTL (19). GO (Gene Ontology, http://www.geneontology.org) and KEGG (Kyoto Encyclopedia of Genes and Genomes, http://www,genome.jp/kegg/) facilitated the functional enrichment analysis of variants-related genes and were performed by KOBAS (http://kobas.cbi.pku.edu.cn/ ).

### Confirmatory experiments

In the original 11 THPT samples and their corresponding normal blood samples, driver mutated genes were selected and analyzed. The mRNAs from these 11 pairs of samples were extracted for reverse transcription, and primers were designed for the target genes. The qRT-PCR was performed to detect the expression differences of six genes, including PRKDC, TBX20, ATAD5, ZNF669, NOX3, and UBR3.

### Statistical analysis methods

Driver gene analysis performed by OncodriveCLUSTL generated three p-values per genomic elements (GEs), including an empirical p-value, an analytical p-value and a second analytical p-value (19). All resulting p-values were adjusted (q-values) using the Benjamini-Hochberg method and GEs below 1% false-discovery rate (FDR) are considered significant. The results shown here were based on ranking of GE score analytical p-values. The driver gene graph, CNV heatmap and Circos graphs were generated using R Studio(version 4.0.2). The result of GO and KRGG analysis were submitted to hypergeometric test and then Fisher’s exact test, and graphical presented by KOBAS. Functional enrichment with p value <0.05 is considered significant.

Clinical baseline data of patients were presented as (Mean ± SD). Statistical analyses of the result of qRT-PCR were performed and graphics were generated using GraphPad Prism (version 9.5.1). Results were presented in graphical format showing (Mean ± SEM) of three repeated experiments. Data conforming to Gaussian distribution were subjected to unpaired t-test. P value <0.05 is considered statistically significant.

## Results

### Clinical information of patients

Our study included 11 Chinese patients, consisting of 4 males and 7 females, who underwent parathyroidectomy for THPT between November 2020 and April 2021. All patients suffered from renal failure, with 9 patients relying on hemodialysis, one patient relying on peritoneal dialysis and one patient having undergone renal transplantation. The average age of the patients was (49.27 ± 6.83) years old, with dialysis duration ranging from 1 to 18 years. Their average preoperative serum calcium level was (2.65 ± 0.11) μmol/L (reference value 2.25-2.4 μmol/L), and average serum phosphorus level (2.01 ± 0.63) mmol/L (reference value 0.81-1.45 mmol/L). The mean preoperative PTH level in 10 uremic patients on dialysis was higher than 1000 pg/mL (reference value 15-65 pg/mL), at the level of (1577.10 ± 537.70) pg/mL, while in the post-transplantation patient, the PTH level was 380 pg/mL. The serum Creatinine level averaged at (822.09 ± 373.36) μmol/L. Nodular hyperplasia or adenomatous hyperplasia of the parathyroid gland was observed in all patients. The detailed baseline characteristics of included patients were provided in **Table 1**.

**Table 1:** Clinical and molecular features of participant patients.

Case	Gender	Treatment	Dialysis duration (year)	Age(years)	Serum PTH Preoperative (pg/mL)	Serum Calcium Preoperative (mmol/L)	Serum phosphorus Preoperative (mmol/L)	Serum Creatinine (μmol/L)
THPT01	F	HD	6	42	1192	2.56	2.47	991
THPT02	F	HD	14	46	1072	2.55	2.35	919
THPT03	M	HD	9	46	2567	2.75	2.43	864
THPT04	F	PD	17	41	2068	2.74	2.41	1031
THPT05	M	HD	1	55	1540	2.65	2.29	1485
THPT06	F	HD	9	57	1209	2.63	2.23	894
THPT07	F	HD	1	43	2294	2.54	1.8	558
THPT08	M	HD	18	57	1286	2.58	1.48	593
THPT09	M	HD	11	43	1105	2.58	1.36	1126
THPT10	F	HD	2	57	1438	2.65	2.68	500
THPT11	F	RT	/	55	380	2.92	0.6	82

M, Male; F, Female; HD, Hemodialysis; PD, Peritoneal dialysis; RT, Renal transplantation.

### Sequencing statistics

The sequencing accuracy was assessed based on the quality value, revealing an average Q30 of 92.90% ± 0.38%. Additionally, the GC content was found to be 48.42% ± 0.80%. A total of 73.32 (range, 56.00 to 100.50) million reads were obtained per tumor sample and 83.26(range, 62.80 to 113.60) million reads per blood sample. The tumor samples exhibited a 73.55% ± 7.35% fraction of genome with 30× coverage and an average median sequencing depth of 45.55×, while the blood samples showed an 81.27% ± 4.97% fraction of genome with 30× coverage and an average median sequencing depth of 53.82×.

### Identification of tumor-specific somatic variants

**Figure 1:**
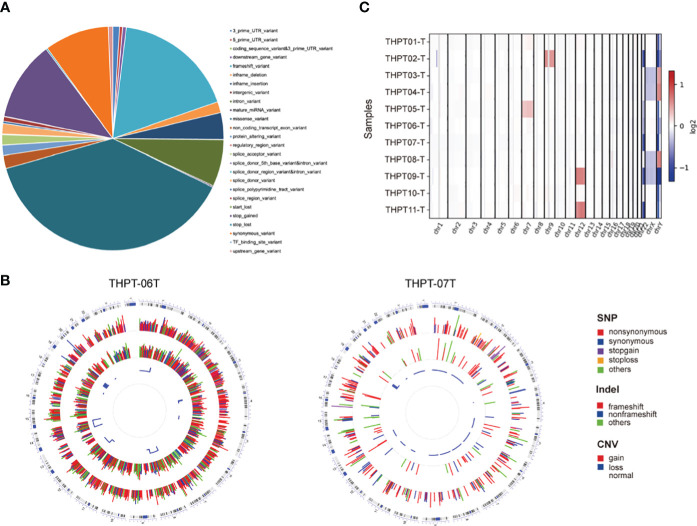
The statistical data of mutation types and distribution on chromosomes in THPT patients. **(A)** The specific proportion of each type of mutation after Quality Control. Missense variants, the most common type, accounted for 38.45% of all the mutations, followed by frameshift variants (17.69%), stop gained (11.52%) and synonymous variants (9.37%). **(B)** Circos plots of the WES data of two patients with the most and fewest mutations. The outermost ring showed the chromosomes, with each chromosome represented as a separate segment of the circle. The second ring showed the SNP along each chromosome, with SNV types represented as colored blocks. The next ring showed Indel, with frameshift, nonframeshift and others as different colored blocks. The innermost ring showed CNV, with gain, loss and normal shown in different colors. **(C)** The distribution of copy number gain and loss in chromosomes of 11 samples. Except for sex chromosomes, copy number gain was observed in Chr12 in THPT tumor sample 09 and 11, and in Chr7 in THPT tumor sample 01 and 05, and in Chr9 in THPT tumor sample 02. Copy number loss was observed in Chr 22 in 6 samples, namely THPT tumor sample 02, 05,06,07 and 11.

According to our filter criteria, a total of 17401 mutations containing 2687 existing known variants were discovered in 11 clinical paired samples, including four most common variants types, 6690 (38.45%) missense variants, 3078 (17.69%) frameshift variants, 2005 (11.52%) stop gained variants and 1630 (9.37%) synonymous variants. A large number of mutations were evenly distributed across the chromosomes, with the exception of the sex chromosomes. The specific proportion of each type of mutation is shown in **Figure 1A**. The detailed distribution of Single Nucleotide Polymorphism (SNP), INDEL, and CNV in the two samples with the most and fewest mutations (4109 and 447) were visualized using Circos and displayed in **Figure 1B**. The distributions in the other nine samples were shown in **Supplementary Figure 1**. Synonymous variants, also known as silent mutations, resulting in no change to the amino acid sequence of a protein, were excluded from further analysis. CNV analysis demonstrated the copy number deletion was observed in chromosome 22 (Chr22) in 6 samples, with loci starting from 15,941,981 to 51,237,548, covering 582 genes. Besides, copy number gain was observed in Chr12 and Chr7 in 2 samples respectively, and in Chr9 in one sample (**Figure 1C**). The detailed CNVs detected in each sample was shown in **Supplementary Figure 2.**

**Figure 2:**
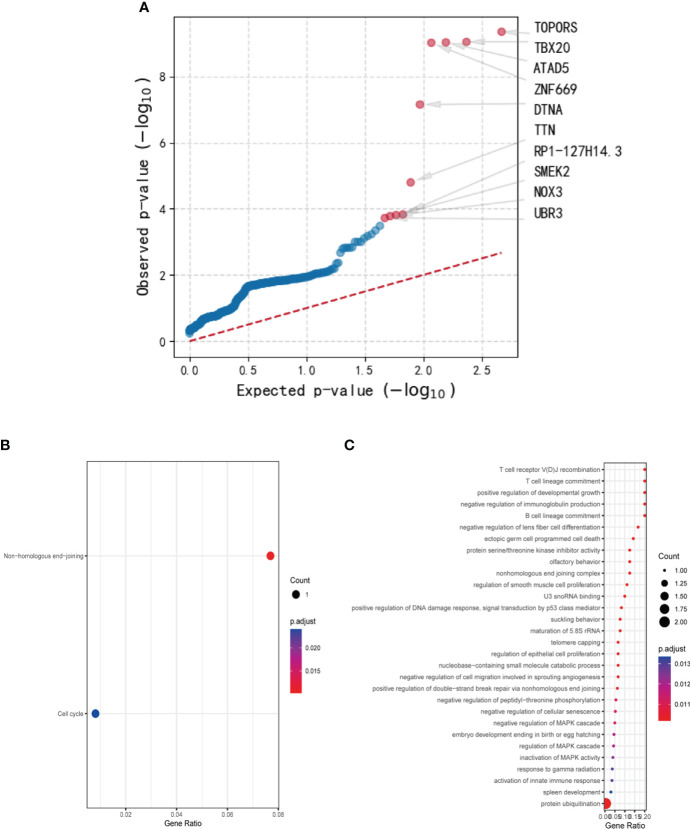
The results of driver mutation analysis and KEGG and GO analyses of the mutated gene set. **(A)** A number of 179 genes emerged as statistically significant during driver mutation analysis. The top 10 significant mutated genes were identified as TOPORS, TBX20, ATAD5, ZNF669, DTNA, TTN, RP1-127H14.3, SMEK2, NOX3, and UBR3. **(B)** Two pathways, non-homologous end-joining and cell cycle, were enriched in KEGG analysis with only one gene involved. **(C)** Various cellular components and cytobiological processes were enriched by GO analysis.

Using genetic mutations that lead to malfunction in the coding region as a background, a number of 179 genes emerged as statistically significant during driver mutation analysis. The top 10 significant mutated genes were identified as TOPORS, TBX20, ATAD5, ZNF669, DTNA, TTN, RP1-127H14.3, SMEK2, NOX3, and UBR3 (**Figure 2A**). Among these mutations, TBX20, ATAD5, ZNF669, and NOX3 were found to have recurrent missense mutations in 3 different patients, respectively.

After conducting Mutect2 analysis and filtering the mutated genes, KEGG and GO analyses were employed to investigate the biological functions and pathways of the identified genes. However, the outcomes of KEGG analysis indicated only two pathways, non-homologous end-joining and cell cycle, which were enriched by a sole gene, PRKDC (**Figure 2B**). In contrast, the GO analysis demonstrated significant enrichment of various cellular components and cytobiological processes, such as positive regulation of developmental growth, protein ubiquitination, and positive regulation of apoptotic process (**Figure 2C**). Interestingly, the GO terms were found to be associated with four genes, namely ENTPD7, PRKDC, SPRED1, and UBR3, signifying their potential role in regulating cellular processes and functions.

The mutation data of the aforementioned genes were exhaustively compiled and summarized in **Table 2**. Specifically, a total of 8 mutations in PRKDC were identified, encompassing recurrent stop gained in exon 21 in 5 samples (5/8), repeated frameshift variant in exon 72 in 2 samples (2/8), and a missense variant in exon 84 in one sample (1/8). The PRKDC mutation was predicted to be one of the foremost driver mutations, ranking 15th in the list. In contrast, a repeated missense variant in exon 3 of ENTPD7 was detected in 5 samples, which was predicted to be tolerant by SIFT and benign by PolyPhen in terms of its effect on the protein sequence. Frameshift variants in 5 samples and missense variants in 2 samples were observed in SPRED1, which were not considered significant in driver gene analysis and thus were eliminated from further consideration. Regarding UBR3, the mutations comprised repeated stop gained in exon 4 in 5 samples and a different stop gained variant in exon 9 in one different sample, frameshift variants in exon 21 in two CDS positions in one sample, and splice donor variants in exon 22 and 15 in 2 samples respectively. It was deemed a significant driver gene in tumors in diver gene analysis, ranking 10th in the results.

**Table 2:** Mutated genes with repeated variants in over 3 samples with detailed mutation information and functional analysis.

Mutated Genes	Variant Types	Exon	AlleleChange	Amino Acid Change	Mutation Frequency	Samples	SIFT Prediction	Polyphen-2 Prediction	Driver gene analysis (Rank)	Function description	
										Enriched GO Terms	PathwaysfromWikipathways
TBX20	Missense	6/8	c.846T>G	p.F282L	3	THPT06, THPT07, THPT08	Deleterious	Probably damaging	2	Not applicable	Heart development Cardiac progenitor differentiation
ZNF669	Missense	2/4	c.331C>G	p.Q111E	3	THPT02, THPT07, THPT11	Deleterious	Benign	4	Not applicable	Not applicable
NOX3	Missense	13/14	c.1582A>T	p.S528C	3	THPT02, THPT04, THPT07	Deleterious	Probably damaging	9	Not applicable	Oxidative stress response; Genetic causes of porto-sinusoidal vascular disease
ENTPD7	Missense	3/13	c.55-56CC>GG	p.P19G	5	THPT02, THPT03, THPT04, THPT07, THPT09	Tolerated	Benign	Not applicable	nucleobase-containing small molecule catabolic process	Not applicable
										endocytic vesicle membrane	
										hydrolase activity	
ATAD5	Missense	8/23	c.2765A>T	p.K922M	3	THPT03, THPT05, THPT08	Deleterious	Benign	3	Not applicable	Not applicable
		10/23	c.3031A>G	p.K1011E	1	THPT06					
		22/23	c.5330G>A	p.S1777N	1	THPT06					
	Frameshift	2/23	c.1018-1019 ccc>cGGGGTATTTTcc	p.P340RGIFX	1	THPT04	Not applicable	Not applicable			
	Stop gained	21/23	c.4778-4779 tcc/tcCTCTGTATCATCTTCCTCAAATGCAGAAGAAAGCAAAACCGGAGACGAAGAAAGCAAAGCCAGTGTCAAATAGATCACTATCATCTAATATTACCAATGTTTTCTTTTGTTTCTTTTTCTGGGACTTTTTCAATGGCTGTTc	p.S1593SSVSSSSNAEESKTGDEESKASVK*ITII*YYQCFLLFLFLGLFQWLF	1	HPT08	Not applicable	Not applicable			
UBR3	Stop gained	14/39	c.2147 T>A	p.L16*	5	THPT02, THPT05, THPT07, THPT10, THPT11	Not applicable	Not applicable	10	suckling behavior	Not applicable
										olfactory behavior	
										protein ubiquitination	
										embryo development	
	Frameshift	21/39	c.2814 atG>at	p.M938X	1	THPT03				ending in birth or egg hatching	
			c.2817-2818->C	p.-939-940X						sensory perception of smell	
	Stop gained & frameshift	9/39	c.1525-1526 act > aCTAACAAGAGGCCAGTAAGTGTTATTCTTCAGTAATGct	p.T509TNKRPVSVILQ*CX	1	THPT08				ubiquitin ligase complex	
										in utero embryonic development	
										ubiquitin-protein transferase activity	
SPRED1	Frameshift	2/7	c.87-94 ggTGGATGGTta>ggta	p.GGWL 29-32GX	5	THPT02, THPT03, THPT04, THPT05, THPT07	Not applicable	Not applicable	433	protein ubiquitination	Microtubule cytoskeleton regulation
										negative regulation of lens fiber cell differentiation	
										protein serine/threonine kinase inhibitor activity	
										positive regulation of DNA damage response, signal transduction by p53 class mediator	
										negative regulation of cell migration involved in sprouting angiogenesis	
			c.95-96 tta>ttAACCATCCa	p.L32LTIX	5					negative regulation of peptidyl-threonine phosphorylation	
										negative regulation of MAPK cascade	
										regulation of MAPK cascade	
										inactivation of MAPK activity	
										negative regulation of epithelial to mesenchymal transition	
	Missense	7/7	c.1112 G>C	p.C371S	2	THPT07 THPT10	deleterious	Probably damaging		phosphatase binding	
										negative regulation of phosphatase activity	
										negative regulation of ERK1 and ERK2 cascade	
										negative regulation of transforming growth factor beta receptor signaling pathway	
			c.1117 G>C	p.E373Q	2					negative regulation of protein kinase activity	
										caveola	
										fibroblast growth factor receptor signaling pathway	
										negative regulation of angiogenesis	
										MAPK cascade	
										cytoplasmic vesicle	
PRKDC	Stop-gained	21/87	c.2337T>A	p.Y779*	5	THPT02, THPT03, THPT04, THPT05, THPT09	Not applicable	Not applicable	15	positive regulation of developmental growth	miRNA regulation of DNA damage response; Cell cycle; Retinoblastoma gene in cancer; Fas ligand pathway and stress induction of heat shock proteins; Pathways affected in adenoid cystic carcinoma; ATM signaling in development and disease; DNA IR-double strand breaks and cellular response via ATM; DNA IR-damage and cellular response via ATR; Non-homologous end joining; DNA damage response; DNA repair pathways, full network
										B cell lineage commitment	
										T cell receptor V(D)J recombination	
										T cell lineage commitment	
										negative regulation of immunoglobulin production	
										ectopic germ cell programmed cell death	
	Frameshift	72/87	c.10087-10088gga/gAATCCTCTGAACTGga	p.G3363ESSELX	2	THPT11	Not applicable	Not applicable		nonhomologous end joining complex	
										regulation of smooth muscle cell proliferation	
	Missense variant	84/87	c.11650C>T	p.R3884W	1	THPT05	deleterious	Probably damaging		U3 snoRNA binding	
										maturation of 5.8S rRNA	
										regulation of epithelial cell proliferation	
										telomere capping	
										positive regulation of double-strand break repair via nonhomologous end joining	
										negative regulation of cellular senescence	
										response to gamma radiation	
										activation of innate immune response	
										T cell differentiation in thymus	
										positive regulation of erythrocyte differentiation	
										spleen development	
										small-subunit processome	
										intrinsic apoptotic signaling pathway in response to DNA damage	
										telomere maintenance	
										protein-DNA complex	
										somitogenesis	
										thymus development	
										response to activity	
										positive regulation of fibroblast proliferation	
										protein destabilization	
										positive regulation of type I interferon production	
										double-strand break repair via nonhomologous end joining	
										double-strand break repair	
										peptidyl-threonine phosphorylation	
										regulation of circadian rhythm	
										rhythmic process	
										regulation of hematopoietic stem cell differentiation	
										negative regulation of protein phosphorylation	
										positive regulation of translation	
										cellular protein modification process	
										double-stranded DNA binding	
										cellular response to insulin stimulus	
										nuclear chromosome, telomeric region	
										peptidyl-serine phosphorylation	
										heart development	
										protein kinase activity	
										transcription regulator complex	
										brain development	
										protein domain specific binding	
										cellular response to DNA damage stimulus	

Based on the comprehensive analyses of above genes, PRKDC and UBR3 stood out as the significant ones that might play a role in carcinogenesis. The expression and mutation of PRKDC and UBR3 in different malignancies were primarily explored and displayed in **Supplementary Figure 3**. Generally, overexpression of PRKDC was observed in most cancers except for renal cancer, in which the level of PRKDC was lower than that in normal tissues (**Supplementary Figure 3A**). On the contrary, the differentiated expression of UBR3 were illustrated to be downregulated in a series of cancers, while merely upregulated in esophageal carcinoma (ESCA) and stomach adenocarcinoma (STAD) (**Supplementary Figure 3B**). Besides, the mutation frequencies of PRKDC in malignancies were dramaticly high, with over 10% of whole tested in uterine corpus endometrial carcinoma (UCEC, 16.57%), skin cutaneous melanoma (SKCM,13.24%), and colon adenocarcinoma (COAD, 10.10%) (**Supplementary Figure 3C**). By contrast, the percentage of cancer samples with UBR3 mutation was relative less, with mutation rate at 9.98% in UCEC being the top one among all the cancers(**Supplementary Figure 3D**).

### The mRNA expression levels of the target genes with mutations

**Figure 3:**
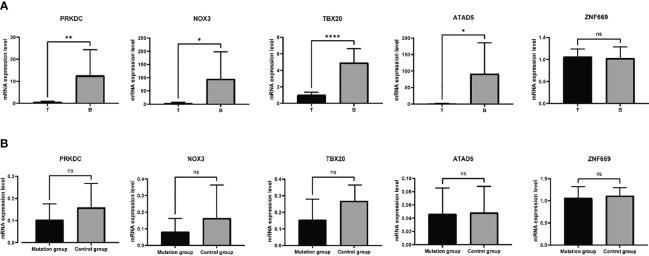
The mRNA expression levels of specific genes in 11 samples tested by qRT-PCR. **(A)** PRKDC, TBX20, ATAD5, and NOX3 were down-expressed in hyperplastic parathyroids compared to normal blood samples, while the expression of ZNF669 was comparable between hyperplastic parathyroids and normal blood samples. **(B)** The expression levels of PRKDC, TBX20, and NOX3 in with exon mutations were relatively lower compared to those without mutations, while the levels of ATAD5 and ZNF669 were unchanged. The difference did not reach statistical significance. *p<0.05, **p<0.01, ****p<0.0001, ns, nonsense, p>0.05.

Based on the aforementioned analyses, a subset of six genes, namely PRKDC, TBX20, ATAD5, ZNF669, NOX3, and UBR3, were selected for further investigation of their mRNA expression levels in 11 samples. Primers designed and applied were illustrated in **Supplementary Table 1**. The qRT-PCR results indicated a general downregulation of PRKDC, TBX20, ATAD5, and NOX3 in hyperplastic parathyroids of THPT compared to blood samples, while the expression of ZNF669 was found to be comparable to that in blood (**Figure 3A**). Additionally, the expression level of UBR3 was so low that qRT-PCR could not detect it. Notably, the expression levels of PRKDC, TBX20, and NOX3 in hyperplastic parathyroids of THPT with exon mutations were relatively lower compared to those without mutations, although the difference did not reach statistical significance (**Figure 3B**).

### Analysis of known mutated genes in sporadic PAs and parathyroid carcinoma

MEN1, a tumor suppressor that are confirmed frequently mutated in sporadic parathyroid adenoma, was detected with no mutation in these THPT samples. Similarly, no mutation was detected in the proto-oncogene of PAs, CCND1, either. Loss-of-function mutations of Cell Division Cycle 73 (CDC73), the tumor suppressor gene playing a role as a major driver genetic defect in the carcinogenesis of parathyroid carcinoma, was observed with a missense mutation in exon 16, predicted to be tolerated and benign in merely one sample in this study. The gene involved in PTH synthesis or regulation pathway, Calcium-sensing Receptor (CaSR), was shown mutated in the form of a novel missense variant in one sample, while it was predicted to be tolerated by SIFT and benign by PolyPhen. Besides, known mutations in PAs revealed via WES analysis, including EZH2, EZH1, ZFX, mTOR and FAT1, were compared with the sequencing data herein. Among these, different variant sites in FAT1 gene were observed in 3 samples, with existing missense variations detected in exon 2 in two distinctive CDS positions in 2 samples. Frameshift variants in exon 11 were observed in ZFX gene in 2 samples. A missense variant in exon 7 in EZH2 was detected in one sample, predicted to be tolerated by SIFT and benign by PolyPhen, and no mutation in exon in EZH1 was detected. A missense variant in exon 4 and 2 frameshift variants in exon 11 in mTOR gene were verified in 2 samples. Further, PI3K-AKT-mTOR pathway, integrating both intercellular and extracellular signals to regulate cell growth, proliferation, metabolism and survival, was also checked. Two existing missense variants in exon 3 were observed in PIK3CA gene in 2 samples. MEP3K1, involved in the Ras-Raf-MEK-ERK pathway, was demonstrated with replicated missense variants in both exon 14 and 17 in 2 different samples respectively.

## Discussion

THPT is a condition characterized by the persistent release of PTH and loss of feedback regulation to blood calcium levels, resulting in hypercalcemia and elevated PTH levels, originally described in 1970s (20, 21). This condition typically arises when treatment for secondary hyperparathyroidism (SHPT) is inadequate, leading to the formation of abnormal parathyroid structures that are unresponsive to medical interventions. Certain gene mutations may contribute to the development and progression of THPT, yet research in this area is limited. In light of this knowledge gap, we sought to conduct a comprehensive investigation of the genetic features of hyperplastic parathyroids in THPT, with the aim of enhancing our understanding of the underlying pathogenesis and molecular characteristics of this condition.

In this study, we examined abnormal parathyroid samples from patients with THPT and found that all displayed adenomatous or nodular hyperplasia as the pathological features. Using WES and CNV analysis, we compared the exome mutations of these hyperplastic parathyroids to corresponding blood samples. After quality control and mutation filtering, we identified a total of 17,401 mutations, with missense variants being the most common, followed by frameshift variants, stop-gained variants, and synonymous variants. Missense, frameshift, and stop-gained variants in genes can alter gene expression and lead to loss or alteration of protein function. Furthermore, recurrent mutations in hyperplastic parathyroids may contribute to the pathogenesis and development of THPT. Following a comprehensive analysis and verification, our study has uncovered three significant genes with variants that are the candidates for driving the pathogenesis of THPT, including PRKDC, TBX20, and NOX3.

Prior researches have endeavored to explicate the molecular characteristics of PHPT stemming from parathyroid adenoma and carcinoma. A prevalent genomic aberration observed in parathyroid adenoma is the loss of heterozygosity of the MEN1 tumor suppressor gene, which governs cell growth and cell cycle. In one study, the incidence of MEN1 mutations was found to reach up to 35% in parathyroid adenoma (22). CCDN1, an oncogene that encodes Cyclin D, has been unequivocally established as a pivotal contributor to the pathogenesis of sporadic parathyroid adenoma and carcinoma (23, 24). Conversely, the inactivation of the tumor suppressor gene CDC73 has been found to be a crucial determinant in the carcinogenesis of parathyroid carcinoma, with a prevalence of up to 70% in sporadic cases (23). In the present study, no recurrent mutations of the aforementioned three genes were detected in hyperplastic parathyroids of THPT, indicating a distinctive driving mechanism of adenomatous hyperplasia of parathyroids in TPHP as compared to PHPT. Additionally, recent research has uncovered somatic mutations in several other genes at a relatively high frequency using WES, including EZH2, EZH1, ZFX, CaSR, and FAT1 (25, 26). While the samples analyzed in this study exhibited only one or two dependent variants with no discernible patterns. The incongruity of gene mutations in hyperplastic parathyroids of THPT and sporadic parathyroid adenoma and parathyroid carcinoma reveals the fundamental disparities of pathogenetic driving genes in THPT, also accounting for the ineffectiveness of medical therapies in treating THPT, despite their definite efficacy in treating PHPT and SHPT. It is noteworthy, however, that mutations in mTOR and PIK3CA genes were identified in two TPHT samples. The overactivity of the PI3K-ATK-mTOR pathway has been confirmed to be involved in reducing apoptosis and promoting proliferation in various cancers, including parathyroid carcinoma (27). The presence of mutations in the mTOR and PIK3CA genes suggests that this renowned intracellular pathway, which regulates the cell cycle, may also contribute to the development or progression of parathyroid adenomatous hyperplasia in TPHT.

To be specific, the mutation frequency of TBX20, NOX3, and PRKDC was equal to or above 3/11. Recurrent missense variants in exon 6 of the TBX20 gene were predicted to be deleterious and damaging by SIFT and Polyphen. We verified that these mutations downregulate the expression of TBX20 mRNA primarily using qRT-PCR. TBX20 is an essential transcription factor in the process of heart development, participating in the regulation of multiple signaling pathways linked to the differentiation and proliferation of cardiac cells. Mutations in this gene have been associated with diverse cardiac defective diseases (28, 29). Among skin cutaneous melanoma cases, TBX20 was detected with missense and splice mutations at a frequency of 5.63% (25 cases), which is the highest alteration frequency among the PanCancer Atlas based on data obtained from the TCGA database. In our study, we found that the expression of TBX20 was positive in hyperplastic parathyroids, although at a lower level than that in blood cells. Samples with mutations expressed relatively lower levels of TBX20, indicating a damaging and negative effect of the mutations on the expression of this gene.

NOX3 is a gene that encodes a member of the NOX family of NADPH oxidases, which play a role in generating superoxide and reactive oxygen species (ROS) and are involved in the oxidative stress response pathway. The gene is generally expressed at low levels in various tissues, except in the inner ear where it is related to the biogenesis of otoconia. In chronic kidney disease, ROS and oxidase stress response were demonstrated to inhibit PTH receptor signaling and trafficking, contributing to the acceleration of osteoporosis (30, 31). Another study probed into the role of PTH in endothelial dysfunction and found that PTH increased ROS and mediated oxidative stress to impair endothelial angiogenic competence *in vitro* experiments (32). NOX3 was found to be dominantly lowly expressed in the hyperplastic parathyroid of THPT compared with blood samples, and samples with missense mutations of the gene expressed relatively lower mRNA levels than those without mutations. The role of NOX3 in generating ROS might explain the remarkably high expression level in blood samples in THPT. Mutations in NOX3 were investigated to be involved in noise-related hearing loss via a Genome-Wide Association Study (33), and the abnormalities in cerebellar structure and function resulting from neuronal precursor cell proliferation induced by ROS in animal models (34). Further investigation is required to determine whether the deleterious mutation of NOX3 in exon 13 plays a role in the hyperplasia of parathyroid and excessive synthesis and secretion of PTH. Additionally, missense mutations in the other two genes, ATAD5 and ZNF669, exerted no effects on gene expression levels, indicating that they were inactive mutations in the exons.

Further, KEGG pathway and GO analyses were performed on a filtered gene set to identify significant enriched pathways and GO terms that shed light on the molecular mechanism of THPT. The results showed that the PRKDC gene played a significant role in various biological processes of the disease. Located on chromosome 8q11.21, the PRKDC gene encodes the DNA-dependent protein kinase catalytic subunit (DNA-PKcs) and is involved in DNA double-strand breaks (DSBs) repair and the maintenance of chromosome stability. Mutations in PRKDC and defects in DNA-PKcs expression have been shown to enhance the process of cell apoptosis due to the generation of DNA DSBs, leading to mutation accumulation and tumorigenesis. Based on TCGA, PRKDC had a high mutation rate in several tumors, including UCEC, SKCM, and COAD, while its expression levels varied in different cancers, as was shown in **Supplementary Figure 2** (35). In colorectal cancer, the mutations in PRKDC was deemed to be one of the primary founder mutations, which leads to increased mutation load, as well as increased tumor heterogeneity (36). In hepatocellular carcinoma, a high mutation frequency (11.5%) was observed in a study cohort, and the matched drugs of DDR-mutant patients were significantly more than those of wild-type patients (37). In this study, recurrent Loss of Function mutations were confirmed in PRKDC in five out of eleven samples, indicating a high mutation frequency in THPT. Furthermore, the expression levels of PRKDC were relatively downregulated in THPT compared to blood samples. Tumor samples with stop-gain mutations in Exon 21 of PRKDC had a lower level in this gene, although the statistical significance was nonsense due to the small sample size and interclass numerical differences. These findings suggest a negative mutation in exon 21 of PRKDC in tumor samples of THPT, which may contribute to the uncontrolled dysplasia of parathyroid by affecting DNA damage repair and cell cycle.

UBR3, ubiquitin protein ligase E3 component n-recognin 3, has been shown to regulate a variety of biological processes through ubiquitination, playing a role in several sensory pathways (38), as well as genetic stability (39). Though the mutation of UBR3 has been detected in several malignancies revealed by the data from TCGA database, few studies delve into the specific role of UBR3 in tumors. Herein, we detected repeated stop gained in exon 4 in 5 samples in UBR3 gene, while the expression level of this gene was too minor to be tested by qRT-PCR. Despite of the predicted role of being the tumor driver gene, the mutation of UBR3 might not contribute dominantly to the pathogenesis of THPT.

What’s more, CNV is a type of genomic variation that alters the copy number of DNA segments in the genome, and it can affect various biological processes, including development, drug response, and disease susceptibility. In this study, CNV analysis revealed frequent copy number deletions in Chr22 among the samples (6/11), providing insights into the susceptibility of chronic kidney dysfunction patients to deteriorating into THPT. Specifically, significant genes located in Chr22 play a role in negative regulation of cell growth or proliferation and act as tumor suppressor genes, such as NF2 (40), CHEK2 (41), and DEPDC5 (42). Deficiencies in these genes, related to CNVs, may contribute to abnormal proliferation of the parathyroid and THPT and warrant further exploration.

In summary, this study presents the genetic characteristics landscape of hyperplastic parathyroids by comparing the genetic profiles of pathological parathyroids of THPT to normal cells. Our findings reveal several distinct genetic underpinnings in THPT, providing novel insights into the pathogenesis and molecular characteristics of THPT. However, in order to fully comprehend the molecular and pathway involved, further functional studies will be necessary in the future.

## Limitations

The findings of this research reveal the presence of several limitations that must be acknowledged. First and foremost, the study’s sample size was limited, which restricted the statistical significance of the results. Additionally, the exclusion of functional studies from the analysis represents a unignored demerit, as such studies *in vitro* and *in vivo* are essential for verifying the roles of the studied variants. By recognizing and addressing these demerits, future research can build upon these findings to arrive at a more comprehensive understanding of the subject.

## Data availability statement

The data presented in the study are deposited in the NCBI repository, accession number PRJNA988842.

## Ethics statement

The studies involving human participants were reviewed and approved by Ethics Review Committee of Naval Medical University. The patients/participants provided their written informed consent to participate in this study.

## Author contributions

Research design: CS; Experiments performing and analysis: LL, QS; Data collection and analysis: QS and HZ; Initial manuscript writing: CS, LL, QW; Manuscript revision: GM, MQ, WL; Final approval of manuscript: CS, WZ. All authors contributed to the article and approved the submitted version.
