# Supplementary material for: Specific genetic aberrations of parathyroid in Chinese patients with tertiary hyperparathyroidism using whole-exome sequencing
Source: Front Endocrinol (Lausanne). 2023 Oct 3;14:1221060. doi: 10.3389/fendo.2023.1221060 (PMC10579901; doi:10.3389/fendo.2023.1221060)

Supplementary Figure1. Detailed distribution of SNV, INDEL and CNV in 9 samples were displayed via Circos.

Suppl.Fig.1

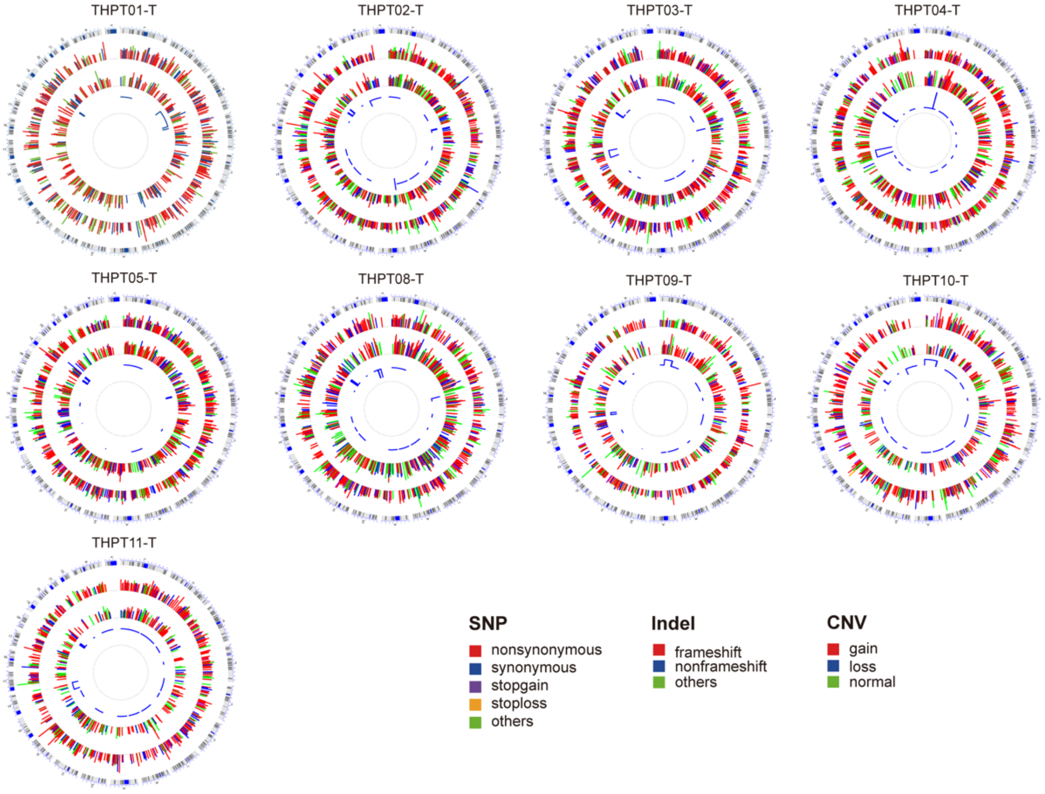

Supplementary Figure2. The detailed CNVs detected in 11 samples.

Suppl.Fig.2

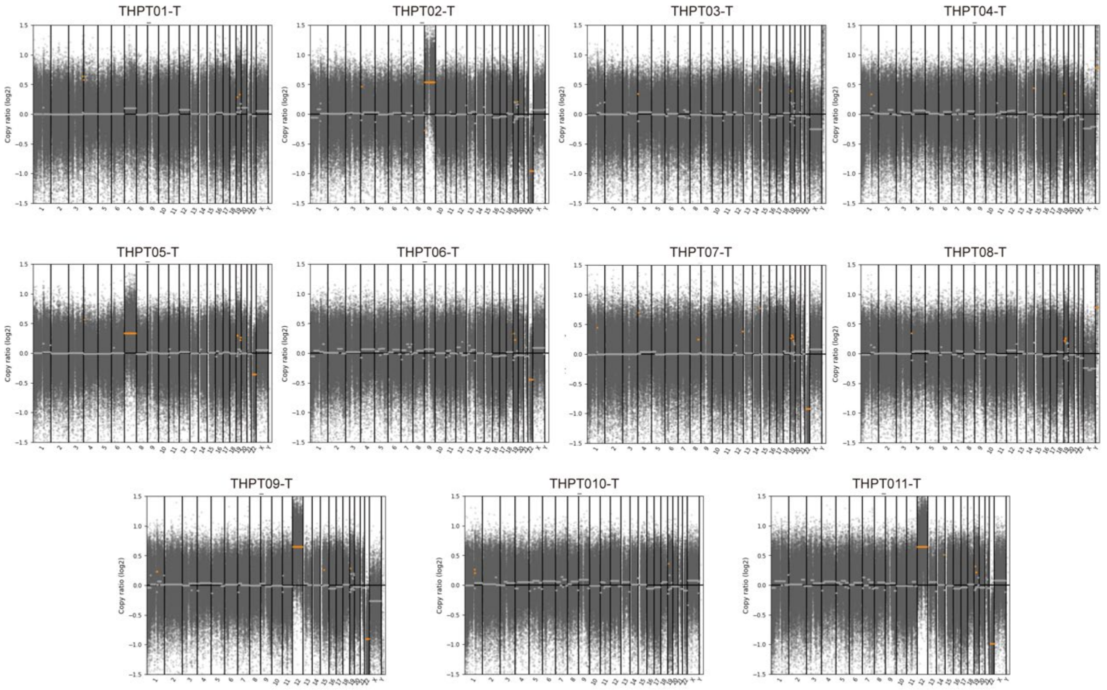

Supplementary Figure3. The expression and mutation ratio of PRKDC and UBR3 in different malignancies obtained from TCGA database.

Suppl.Fig.3

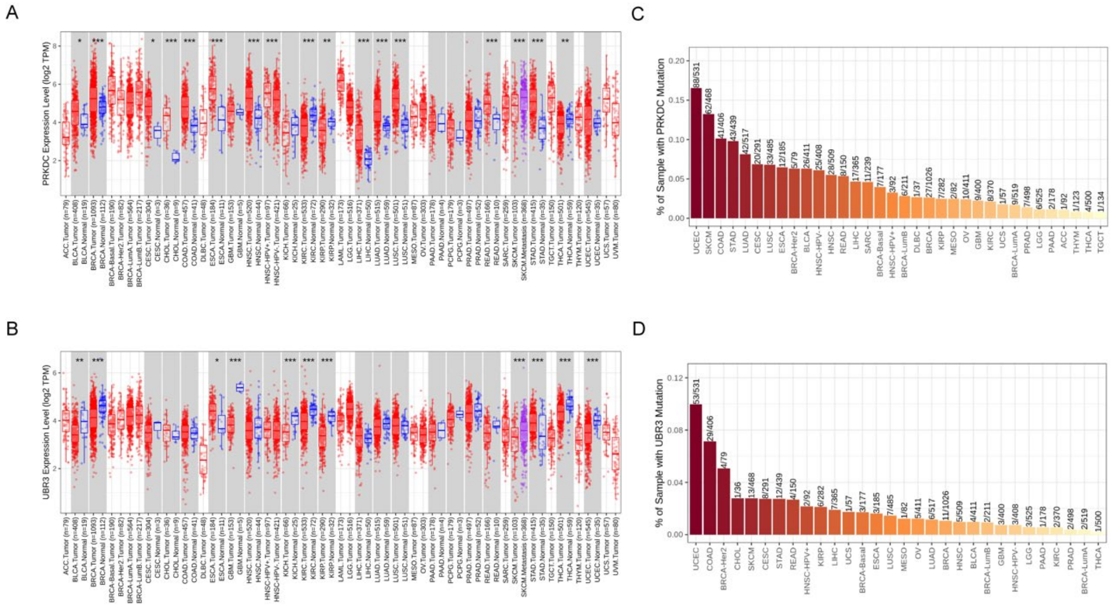

Supplement: Supplementary file 1 [file Image_1.pdf]
