# Supplementary material for: Specific genetic aberrations of parathyroid in Chinese patients with tertiary hyperparathyroidism using whole-exome sequencing
Source: Front Endocrinol (Lausanne). 2023 Oct 3;14:1221060. doi: 10.3389/fendo.2023.1221060 (PMC10579901; doi:10.3389/fendo.2023.1221060)
Supplement: Supplementary file 2 [file Table_1.docx]

Supplementary Table1. The data of primers of target genes and Internal reference gene.

| Gene | Forward-Primer | Reverse-primer | Amplification fragment size(bp) |
| --- | --- | --- | --- |
| PRKDC | CCTGGCCGGTCATCAACTG | AGTAAGGTGCGATCTTCTGGC | 227 |
| TBX20 | ATTCCTATGCACGCTCACCC | GTTGTAAAGGCTGACCCTCG | 100 |
| ATAD5 | GTGAAGGACTGCGAGATTGAG | TGTCTCTAGTCTTCCCTAGTGGT | 109 |
| ZNF669 | GCCTTTAGTCGTTTGAGTTCCC | ATGGCGAGTAAGGTGACTGGA | 201 |
| NOX3 | ACACCCCTAACGAGAGCTACC | TGACGCCTGCTATTGTCCTTA | 87 |
| UBR3 | TTTGGAGGATCACGGTTTGTTAG | GCTCGTTTAGTTCTCGCTTGTTT | 201 |
| GAPDH | TGACTTCAACAGCGACACCCA | CACCCTGTTGCTGTAGCCAAA | 121 |
